# Comparison of Artificial Neural Network and Logistic Regression Models for Predicting In-Hospital Mortality after Primary Liver Cancer Surgery

**DOI:** 10.1371/journal.pone.0035781

**Published:** 2012-04-26

**Authors:** Hon-Yi Shi, King-Teh Lee, Hao-Hsien Lee, Wen-Hsien Ho, Ding-Ping Sun, Jhi-Joung Wang, Chong-Chi Chiu

**Affiliations:** 1 Department of Healthcare Administration and Medical Informatics, Kaohsiung Medical University, Kaohsiung, Taiwan; 2 Department of Surgery, Kaohsiung Medical University Hospital, Kaohsiung, Taiwan; 3 Department of Surgery, Chi Mei Medical Center, Liouying, Taiwan; 4 Department of Surgery, Chi Mei Medical Center, Tainan, Taiwan; 5 Department of Medical Research, Chi Mei Medical Center, Tainan, Taiwan; 6 Department of Surgery, Taipei Medical University, Taipei, Taiwan; 7 Department of Cosmetic Science, Chia Nan University of Pharmacy and Science, Tainan, Taiwan; University of North Carolina School of Medicine, United States of America

## Abstract

**Background:**

Since most published articles comparing the performance of artificial neural network (ANN) models and logistic regression (LR) models for predicting hepatocellular carcinoma (HCC) outcomes used only a single dataset, the essential issue of internal validity (reproducibility) of the models has not been addressed. The study purposes to validate the use of ANN model for predicting in-hospital mortality in HCC surgery patients in Taiwan and to compare the predictive accuracy of ANN with that of LR model.

**Methodology/Principal Findings:**

Patients who underwent a HCC surgery during the period from 1998 to 2009 were included in the study. This study retrospectively compared 1,000 pairs of LR and ANN models based on initial clinical data for 22,926 HCC surgery patients. For each pair of ANN and LR models, the area under the receiver operating characteristic (AUROC) curves, Hosmer-Lemeshow (H-L) statistics and accuracy rate were calculated and compared using paired T-tests. A global sensitivity analysis was also performed to assess the relative significance of input parameters in the system model and the relative importance of variables. Compared to the LR models, the ANN models had a better accuracy rate in 97.28% of cases, a better H-L statistic in 41.18% of cases, and a better AUROC curve in 84.67% of cases. Surgeon volume was the most influential (sensitive) parameter affecting in-hospital mortality followed by age and lengths of stay.

**Conclusions/Significance:**

In comparison with the conventional LR model, the ANN model in the study was more accurate in predicting in-hospital mortality and had higher overall performance indices. Further studies of this model may consider the effect of a more detailed database that includes complications and clinical examination findings as well as more detailed outcome data.

## Introduction

Hepatocellular carcinoma (HCC) is prevalent in regions of Asia, the Mediterranean, and South Africa. In Taiwan, a Hepatitis B virus (HBV) and Hepatitis C virus (HCV) epidemic region, HCC is the leading cause of cancer deaths in males [1]. The incidence of HCC has also increased in the both United States and the United Kingdom in the past two decades [1]–[3]. Prognosis is usually dismal, and the only known curative therapies are surgical, i.e., hepatic resection or liver transplantation. Additionally, the percentage patients with appropriate indications for surgery are relatively small [2]. In recent years, studies of surgical treatment for HCC and other diseases have attempted to develop models for predicting surgical outcome [4]–[6]. However, outcome prediction models with acceptable accuracy have been difficult to develop [7].

Artificial neural networks (ANNs) are complex and flexible nonlinear systems with properties not found in other modeling systems. These properties include robust performance in dealing with noisy or incomplete input patterns, high fault tolerance, and the ability to generalize from the input data [8], [9]. Although many different ANNs have been developed, a common feature is an interconnected group of nodes in multiple layers, in which input nodes and output nodes have clinical correlates [10]. Hidden nodes, which connect to inputs and outputs, allow nonlinear interactions among the input variables and do not have real-world correlates. The nodes are connected by links, each of which has an associated weight. This network is “trained” by exposure to inputs paired with known outputs, and learning occurs when the weights between nodes are modified according to feedback [8]–[10]. The computational power of an ANN is derived from the distributed nature of connections. Once a model is trained, prediction outputs can be generated from novel records [8]–[10].

Previous comparisons of logistic regression (LR) and ANN models for predicting outcomes of HCC surgery have shown major shortcomings [11], [12]. Firstly, few have used longitudinal data for more than two years. Secondly, the data used in most studies have been for HCC patient populations in the United States or in Organization for Economic Co-operation and Development (OECD) countries, which may substantially differ from those in Taiwan. Thirdly, no studies have considered group differences in other factors such as age, gender and nonsurgical treatment. Finally, since most published articles comparing the performance of ANN models and LR models for predicting HCC outcomes used only a single dataset, the essential issue of internal validity (reproducibility) of the models has not been addressed.

Therefore, the primary aim of this study was to validate the use of ANN models for predicting in-hospital mortality in HCC surgery patients. The secondary aim was to compare outcome prediction between ANN and LR models.

## Materials and Methods

### Ethics Statement

This study analyzed administrative claims data obtained from the Taiwan Bureau of National Health Insurance (BNHI). Because the BNHI is the sole payer in Taiwan, the BNHI data set was assumedly the most comprehensive and reliable data source for the study. The subjects of this study were recruited by reviewing monthly patient discharge data released by the BNHI. Furthermore, the database contains a registry of contracted medical facilities, a registry of board-certified physicians and monthly summaries for all inpatient claims. Because these were aggregate secondary data without personal identification, this study was exempt from full review by the internal review board. The study protocol conforms to ethical standards according to the Declaration of Helsinki published in 1964. Additionally, the requirement for written or verbal patients' consent for this data linkage study was waived.

### Study Population

The study sample included all patients diagnosed with malignant neoplasm of liver and intrahepatic bile ducts (ICD-9-CM codes 155.XX) during the years 1998–2009 (n = 148,018). After excluding cases other than those who had received partial hepatectomy (ICD-9-CM procedure code 50.22) or liver lobectomy (ICD-9-CM procedure code 50.3), 24,748 cases remained. Patients with secondary and unspecified malignant neoplasm (ICD-9-CM codes 196.XX–199.XX), malignant neoplasm of intrahepatic bile ducts (ICD-9-CM code 155.1), or malignant neoplasm of the liver other than a primary or secondary neoplasm (ICD-9-CM code 155.2) were also excluded, which left a sample of 22,926 eligible subjects with primary liver malignancy who had received hepatectomies during the study period.

### Potential Confounders

The analyzed patient characteristics and hospital characteristics of the study population included age, gender, co-morbidity, hospital volume, surgeon volume, length of stay (LOS), and in-hospital survival. Co-morbidity was estimated using the Charlson co-morbidity index (CCI) [13]. For each hospital or surgeon, HCC volume was defined by calculating the percentage of HCC surgeries in the total surgeries performed by the respective hospital or surgeon during the study period. Specifically, HCC volume for a hospital or surgeon was categorized as low, medium, high, and very high if the number of HCC surgeries performed by the hospital or surgeon during a given year in the study period comprised <$>\raster="rg1"<$>25%, 26%∼50%, 51%∼74%, and <$>\raster="rg2"<$>75%, respectively, of the total surgical procedures performed by the hospital or surgeon that year.

### Development of the LR model

The dataset was randomly divided into a training set of 18,341 cases (80% of the overall dataset) and a test set of 4,585 cases (20% of the overall dataset). The training set was used to build the LR model. Age, gender, CCI, hospital volume, surgeon volume and LOS were the independent variables, and outcome (death/survival) was the dependent variable. The LR model was then tested using the testing dataset. These steps (randomized division of dataset and regression analysis considering the same variables) were repeated 1,000 times to obtain 1,000 pairs of training and testing datasets (80% and 20% of the original dataset, respectively), which were saved for further processing by the neural network.

### Development of the ANN model

The ANN used in this study was a standard feed-forward, back-propagation neural network with three layers: an input layer, a hidden layer and an output layer. The multilayer perceptron (MLP) network is an emerging tool for designing special classes of layered feed-forward networks [14]. Its input layer consists of source nodes, and its output layer consists of neurons; these two layers connect the network to the outside world. In addition to these two layers, the MLP usually has one or more layers of neurons referred to as hidden neurons because they are not directly accessible. The hidden neurons extract important features contained in the input data.

An MLP is usually trained by a back-propagation (BP) algorithm with forward and backward phases [14]. The BP learning algorithm is easily implemented, and its linear complexity in the synaptic weights of the network makes it computationally efficient. For optimal learning efficiency, the neurons are usually activated with both anti-symmetric functions (e.g., hyperbolic tangent function) and non-symmetric functions (e.g., logistic function). The following cross-validation technique is used to optimize the time when a MLP network training session “stops”. First, one estimation subset of the examples is used for model training, and one validation subset is used for evaluating model performance. The neural network is optimized using a training data set. A separate test data set is used to halt training to mitigate over-fitting. The training cycle is repeated until the test error no longer decreases [15], [16].

**Table 1:** Patient characteristics and hospital characteristics (N = 22,926).

Variable	No. of patients (%)
Operation years	
1998–2001	6,857 (29.9)
2002–2005	7,245 (31.6)
2006–2009	8,824 (38.5)
Age [mean ± standard deviation], years	58.6±12.7
Gender	
Female	6,028 (26.3)
Male	16,898 (73.7)
Charlson co-morbidity index [mean ± standard deviation], scores	3.6±1.6
Hospital volume	
Low	4,218 (18.4)
Medium	6,145 (26.8)
High	6,086 (26.6)
Very high	6,477 (28.2)
Surgeon volume	
Low	5,250 (22.9)
Medium	5,860 (25.6)
High	5,806 (25.3)
Very high	6,010 (26.2)
Length of stay [mean ± standard deviation], days	17.8±9.7
In-hospital mortality	
Survival	22,307 (97.3)
Death	619 (2.7)

**Table 2:** The LR model using selected variables related to in-hospital mortality.

Variable	Un-standardized coefficient	Standardized error	Odds ratio (OR)	P value
Age	−0.042	0.005	1.04	<0.001
Gender^a^				
Male	−0.213	0.054	1.24	0.002
Charlson co-morbidity index	−0.208	0.027	1.23	<0.001
Hospital volume^a^				
Medium	0.284	0.131	1.13	<0.001
High	0.660	0.265	1.52	<0.001
Very High	0.719	0.273	1.84	<0.001
Surgeon volume^a^				
Medium	0.659	0.143	1.22	<0.001
High	0.937	0.155	1.79	<0.001
Very High	1.549	0.215	2.41	<0.001
Length of stay	−0.039	0.004	1.04	<0.001
Constant	7.267	0.355	2.01	<0.001

LR = logistic regression.

a Reference variables are female gender, low hospital volume, low surgeon volume.

### Statistical analysis

The unit of analysis in this study was the individual HCC surgical patient. The data analysis was performed in several stages. Firstly, continuous variables were tested for statistical significance by one-way analysis of variance (ANOVA), and categorical variables were tested by Fisher exact analysis. Univariate analyses were performed to identify significant predictors (p<0.05). Secondly, the discriminatory power of the models was analyzed using area under the receiver operating characteristic curves (AUROCs). Here, discriminatory power refers to the ability of a model to distinguish those who died from those who survived. A perfectly discriminating model would assign a higher probability of death to patients who died than to patients who survived. Thirdly, the relative calibration of the models was compared using the Hosmer-Lemeshow (H-L) statistic to study the predictive accuracy of the models over the entire range of severity. The H-L statistic is a single summary measure of calibration and is based on a comparison of observed and estimated mortality in patients grouped by estimated mortality [17]. The lower the H-L statistic, the better the fit. Therefore, a perfectly calibrated model should have an H-L value of zero. Finally, sensitivity analysis was performed to assess the importance of variables in the fitted models. To simplify the training process, key variables were introduced, and unnecessary variables were excluded. A sensitivity analysis was also performed to assess the relative significance of input parameters in the system model and to rank the importance of the variables. The global sensitivity of the input variables against the output variable was expressed as the ratio of the network error (sum of squares of residuals) with a given input omitted to the network error with the input included. A ratio of 1 or lower indicates that the variable diminishes network performance and should be removed.

**Figure 1:**
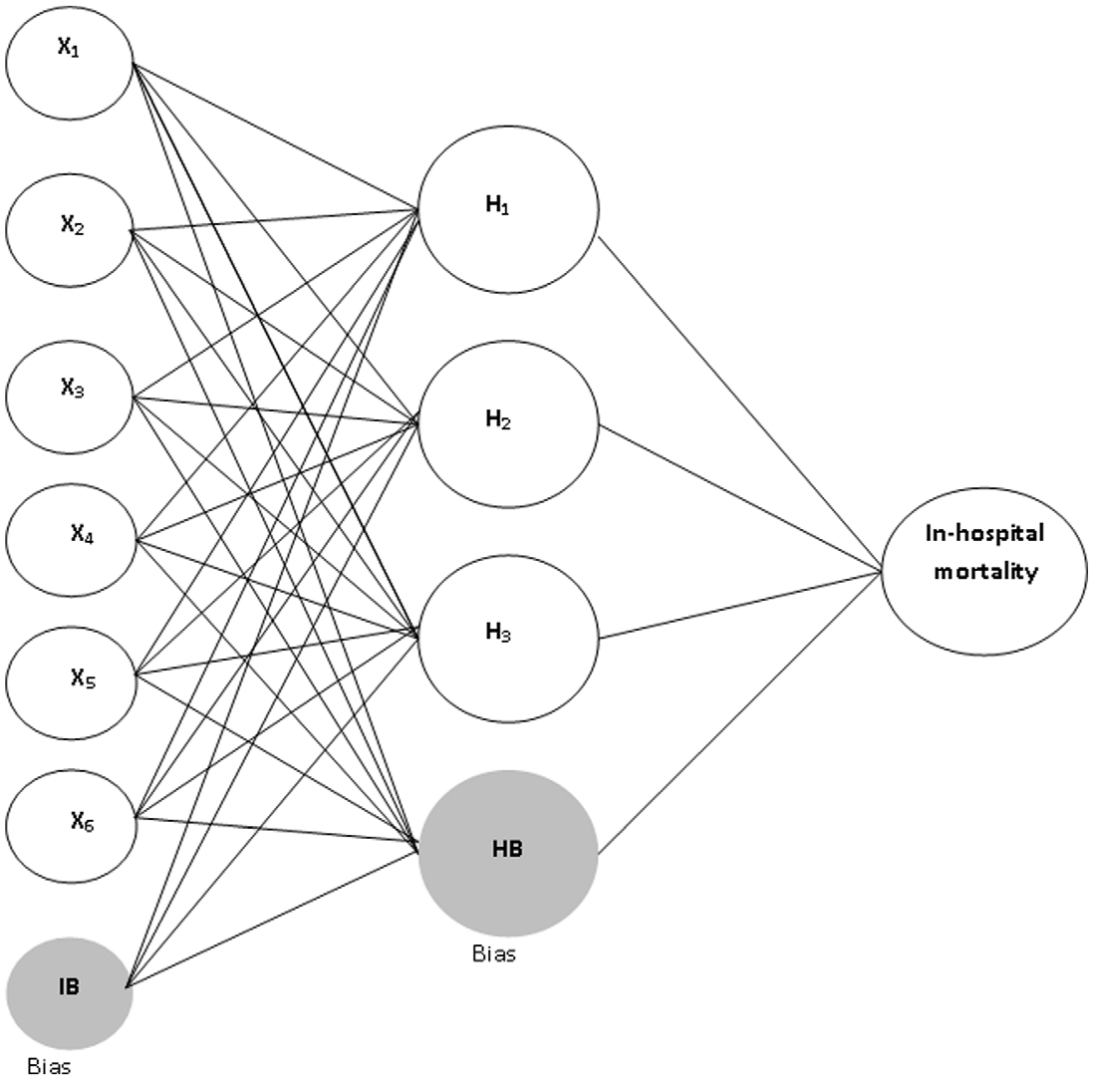
Schematic representation of artificial neural network model with 6 input nodes, 3 nodes in a single hidden layer, and a single output node representing in-hospital mortality. X_1_, age; X_2_, gender; X_3_, Charlson co-morbidity index; X_4_, hospital volume; X_5_, surgeon volume; X_6_, length of stay; IB, input layer bias; HB, hidden layer bias.

For every 1000 pairs of ANN models and LR models (trained and tested on the same datasets) these indices (accuracy rate, AUROC, and H-L statistic) were calculated and compared using paired T-tests.

The STATISTICA 10.0 (StatSoft, Tulsa, OK) software was used to construct the ANN models and LR models of the relationship between the identified predictors and selected significant variables (p<0.05).

## Results

Table 1 shows the patient characteristics and hospital characteristics of the study. The mean age of the study population was 58.6 years (standard deviation 12.7), and 73.7% of the patients were male. The overall in-hospital mortality rate was 97.3%. The mean CCI in the study population was 3.6 (standard deviation 1.6). Table 2 shows the coefficients for in-hospital mortality obtained for the training set in the LR model. In-hospital mortality had a significant negative association with age, male gender, CCI and LOS (p<0.05) but a significant positive association with hospital volume and surgeon volume (p<0.05).

The ANN-based approaches used 3-layer networks and the relative weights of neurons to predict in-hospital mortality. The MLP model included 6 inputs (i.e., age, gender, CCI, hospital volume, surgeon volume, and LOS), 1 bias neuron in the input layer, 3 hidden neurons, 1 bias neuron in the hidden layer, and 1 output neuron (Figure 1). The activation functions of logistic sigmoid and hyperbolic tangent are used in each neuron of the hidden layer and output layer, respectively.

Table 3 shows that ANN significantly outperformed LR in terms of discrimination, calibration, and accuracy (cutoff point 0.5). Compared to LR, ANN had a superior accuracy rate in 97.28% of cases, a superior H-L statistic in 41.18% of cases, and a superior AUROC in 84.67% of cases.

**Table 3:** Comparison of 1000 pairs of ANN and LR models for predicting in-hospital mortality.

Performance indices	ANN (95% C.I.)	LR (95% C.I.)	P value
Accuracy rate	97.28 (95.88, 98.68)	88.29 (86.49, 90.09)	<0.001
H-L statistics	41.18 (34.67, 47.68)	54.53 (49.53, 59.52)	<0.001
AUROC	0.84 (0.88, 0.80)	0.76 (0.71, 0.81)	<0.001

ANN = artificial neural network; LR = logistic regression; Hosmer-Lemeshow statistics = H-L statistics; AUROC = area under the receiver operating characteristic.

The training data set was also used to calculate the variable sensitivity ratios (VSR) for the ANN model. Table 4 shows the VSR values for the outcome variable (in-hospital mortality) in relation to gender, age, CCI, hospital volume, surgeon volume and LOS. In the ANN model, surgeon volume was the most influential (sensitive) parameter affecting in-hospital mortality followed by age and LOS. All VSR values exceeded 1, which indicated that the network performed better when all variables were considered.

**Table 4:** Global sensitivity analysis of the ANN model in predicting in-hospital mortality.

	Rank
	First	Second	Third
Variable	Surgeon volume	Age	Lengths of stay
VSR	1.22	1.10	1.09

ANN = artificial neural network; VSR = variable sensitivity ratio.

Table 5 compares the ANN model and LR model in terms of sensitivity, specificity, positive predictive value (PPV), negative predictive value (NPV), accuracy rate, and AUROC. Together, these values confirmed that the ANN model had superior sensitivity (78.40% versus 62.64%), specificity (94.57% versus 91.92%), PPV (84.22% versus 76.65%), NPV (96.91% versus 87.18%), accuracy rate (95.93% versus 84.47%) and AUROC (0.82 versus 0.73).

**Table 5:** Comparative performance indices of ANN and LR models when using 100 new data sets to predict in-hospital mortality.

Model	Sensitivity (%)	Specificity (%)	PPV (%)	NPV (%)	Accuracy rate (%)	AUROC
ANN	78.40	94.57	84.22	96.91	95.93	0.82
LR	62.64	91.92	76.65	87.18	84.47	0.73

ANN = artificial neural network; LR = logistic regression; PPV = positive predictive value; NPV = negative predictive value; AUROC = area under the receiver operating characteristic.

## Discussion

The comparison of prediction models in this study showed that accuracy in predicting in-hospital mortality was significantly higher in the ANN model than in the LR model (p<0.001). To our knowledge, this study is the first to use a nationwide population-based database to train and test a neural network for predicting HCC surgery outcome. The neural network model was compared with actual outcomes and with an LR model constructed using identical inputs. Given a limited number of clinical inputs and a specific outcome measure, the ANN model consistently outperformed the LR model.

Whereas other prediction models have used data for a single medical center, the prediction model in this study was constructed using national registry data from the Taiwan BNHI. Therefore, it gives a better overview of current outcomes of HCC surgery in an HBV and HCV epidemic region. Compared to data obtained by single-center series studies, data from registry studies provide a better overview of practices in large populations while avoiding referral bias or bias reflecting the practices of individual surgeons or institutions [18], [19].

Because ANNs use a dynamic approach to analyzing mortality risk, they can modify their internal structure in relation to a functional objective by bottom-up computation (i.e., by using the data themselves to generate the model). Although they cannot deal with missing data, ANNs can simultaneously handle numerous variables by building models with reference to outliers and nonlinear interactions among variables [8]–[10]. Whereas conventional statistical methods reveal parameters that are significant only for the overall population, ANNs include parameters that are significant at the individual level even if they are not significant for the overall population. Unlike other standard statistical tests, ANNs can also manage complexity even when the sample size is small and even when the ratio between variables and records is unbalanced [8]–[10]. That is, ANNs avoid the dimensionality problem. The large and homogeneous dataset in this study enabled robust network training because all clinical variables had shown potential impacts on mortality in previous LR models [7], [20].

Chen et al. showed that ANN combined with genetic algorithm can identify clinically significant variables and can precisely predict Tacrolimus blood concentrations in liver transplantation patients [21]. In a comparison of ANN and LR models for predicting cirrhosis in chronic hepatitis C patients, Cazzaniga et al. also showed that the ANNs were slightly more accurate and more reproducible [20]. Recently, Cucchetti et al showed that ANN is more accurate than conventional LR for identifying HCC tumor grade and microscopic vascular invasion based on preoperative variables and is preferable to LR for tailoring clinical management [5].

The ANN approach developed in this study extends the predictive range of the LR model by replacing identity functions with nonlinear activation functions. The approach is apparently superior to linear regression for describing systems. The ANNs may be trained with data acquired in various clinical contexts and can consider local expertise, racial differences, and other variables with uncertain effects on clinical outcome [8]–[10]. The analysis is not limited to clinical parameters. Other variables could be tested for use in improving the predictive accuracy of the model. The proposed ANN architecture can also include more than one dependent variable and can perform a non-linear transformation between dependent variables. Future studies may evaluate how other patient characteristics or clinical characteristics affect the proposed architecture.

Throughout this nationwide population-based study, the best single predictor of in-hospital mortality was surgeon volume, which was consistent with the results of other reports that high-volume surgeons consistently achieve superior outcomes of hepatectomy for HCC [22], [23]. Therefore, their treatment strategies should be carefully analyzed and emulated. If in-hospital mortality is considered a benchmark, surgeon volume, which is a major predictor of postoperative outcome, is crucial. Clearly, outcomes of surgical procedures depend not only on patient management, but also on the skill and experience of individual surgeons. Meanwhile, high-volume surgeons in high-volume hospitals are most likely to achieve good patient outcomes because they are assisted by highly skilled and interdisciplinary care teams [22], [23].

This study has several limitations that are inherent in any large database analysis. Firstly, the clinical picture obtained in this analysis of claims data is not as precise as that of a prospective analysis of clinical trial data due to possible errors in the coding of primary diagnoses and surgical modalities. Secondly, complications associated with HCC surgical procedures were not assessed, which limits the validity of the comparison. Finally, only LR and ANN models were used to predict in-hospital mortality after HCC surgery. The database could not be used to predict other outcomes such as patient-reported quality of life. However, given the robust magnitude of the effects and the statistical significance of the effects observed in this study, these limitations are unlikely to compromise the results.

In conclusion, compared with the conventional LR model, the ANN model in this study was more accurate in predicting in-hospital mortality and had higher overall performance indices. The global sensitivity analysis also showed that surgeon volume was the best predictor of in-hospital mortality after HCC surgery. The predictors analyzed in this study could be addressed by healthcare professionals during preoperative and postoperative health care consultations with candidates for HCC surgery to educate them in the expected course of recovery and health outcomes. Further studies of this model may consider the effect of a more detailed database that includes complications and clinical examination findings as well as more detailed outcome data. Hopefully, the model will evolve into an effective adjunctive clinical decision making tool.
